# Proceedings of Am. Dental Convention

**Published:** 1864-11

**Authors:** 


### PROCEEDINGS OF AM. DENTAL CONVENTION.

(*Continued from Page* 467.)

appears, enucleating, by means of a properly-shaped blunt instrument or spud. He mentioned the case of Brignoli, the singer, whom he treated for the denudation of the palatine fang of an upper molar, with the result of covering its sides one-third of the way down, and having an encouraging prospect of the almost entire reproduction of the alveolus.

Dr. Buckingham thought that the treatment of syphilis did not come within the province of the dentist, but believed that the subject, in its selection, had reference more particularly to the absorption of the alveolar border after extraction, by the natural process.

The hour having arrived, the choice of the next place of meeting was made the order of business, when the Convention, with but little detention, unanimously pronounced in favor of White Sulphur Springs, Ohio.

Dr. Kulp offered the following :—

*Whereas*, The President of the United States has appointed Thursday, August 4, as a day of fasting and prayer; be it therefore

*Resolved*, That this Convention observe the day by suspending the regular order of business from ten to half-past eleven A. M. to morrow, and that the Chairman appoint a committee of three to arrange an appropriate programme. Carried.

In accordance with this resolution, the Chair appointed Drs. Kulp, Taft, and S. S. White. Adjourned.

SECOND DAY.—*Afternoon Session.*

Called to order at three P. M. Minutes read and approved.

Dr. Perkins referred to the persistent spontaneous separation of the teeth occasionally observed and producing a very unsightly appearance.

Dr. Taft believed that almost every case of alveolar absorption was due to systemic influence or influences; the local cause, however, sometimes consisting of a deposition of tartar or inflammatory action in the gum. He thought what was termed absorption was frequently nothing more than a simple solution and washing away of the alveolar tissue-Defective nutrition would often be found to be the cause of such difficulties, arising either from too little food, a misappropriation of that presented, or the presence of poisonous or contaminating influences in the blood, and calling for the administration of constitutional remedies before or in connection with topical treatment. To treat cases intelligently, he believed it indispensable to possess the power of appreciating individual peculiarities. He said that where teeth move and remain apart, there is an absorption of alveolar structure upon one side, and a corresponding deposition upon the other; but in relation to the cause of such separation he was unable to pronounce.

Dr. Atkinson said that the gum wastes from a want of that support which the alveolar tissue had previously afforded.

Upon motion, the next subject, "Filling Teeth: the relative value of different materials, and the mode of operating in difficult cases," was taken up.

Dr. Perkins spoke of the necessity of cutting out all sulci surrounding a cavity, even where they extend entirely over to the margin of the gum.

Dr. J. Ward Ellis indorsed this practice of Dr. Perkins. He would like to hear the various methods employed by gentlemen for keeping the lower teeth dry during filling, as this was a point which had occasioned him more than a little annoyance, sometimes compelling the reluctant performance of a submarine operation.

Dr. Perkins holds his napkins in place with the fingers, and changes them as often as requisite, without any difficulty. He spoke of a case in which he had satisfactorily prevented the annoyance from hæmorrhage of the gum by a soft pine wedge, driven in well up between the necks of the teeth.

Dr. Whitney uses wedges of wood between the teeth in filling, but does not force them in with the mallet.

Dr. Bishop also introduces wood between the teeth by hand force, but in some cases makes use of cotton twine.

Dr. Allport, in order to check the flow of saliva, first wipes the mouth dry with cloths, and then places rolls of bibulous paper over the orifices of the ducts, covering these again with a napkin, which he retains in position by his fingers, never having employed a tongue-holder of any description. In approximal cavities approaching the margin of the gum, he almost invariably introduces a wedge, being particular to insert it below the edge of the cavity, although a little pain should be induced. He regarded *precision* and *rapidity* of manipulation as a most valuable combination, and did not consider *slowness* as at all requisite for the performance of a good operation.

Dr. Forbes said that when one hour had been expended in the preparation of a difficult lower cavity, he would dismiss the patient, and complete the filling at another sitting, thus avoiding the annoyance occasioned by restlessness and a profuse flow of saliva provoked by lengthy manipulation. He fills with cylinders; and in lower approximal cavities first forces a large one down upon the bottom, which, being allowed to project beyond the edge, prevents the saliva from finding ready ingress at that border. He always excavates and fills the fissures alluded to by Dr. Perkins; yet thought that they would sometimes occur after the performance of an operation, which fact should prompt us to be charitable in our opinions of others.

Dr. W. H. Allen uses adhesive foil, heating each pellet in the flame of a spirit-lamp before its introduction, and builds up from the retaining points out with serrated instruments and the mallet. In weak, frail teeth he fills so as to have the gold hold and support the tooth. He fills very small approximal cavities in front teeth with crystal gold. To prevent the seeping of mucus from the margin of the gum, he employs the method suggested by Dr. Metcalf, of encircling the tooth with a string loosely wrapped with foil, which may, by gentle pressure, be sufficiently well adapted to the necks of the teeth.

Dr. Atkinson said that any man who does not make the tooth and filling reciprocally support each other, can be classed no higher than a second-rate operator. He gains all the space desired by properly shaping a wedge, rounding its corners and driving it between the teeth with one, two, or three taps of the mallet, and discountenances the wedging of teeth over night, or for any time longer than that consumed in the operation of filling. Having obtained the desired separation, he prepares the cavity, bevels the edges, fills overlapping, files off the surplus of gold, rasps down with corundum tape, and burnishes upon the surface a ribbon of five, six, or eight layers of No. 6 foil, which gives an even and beautiful finish. When teeth are loose and movable, he supports with wedges during the introduction of a filling. He said that the virtue and perfection of a filling depend upon its accurate adaptation and power of excluding moisture and all foreign materials, and thought that the discoloration of soft foil fillings was owing to their imperfect packing.

Dr. Peirce believed that by the use of cotton and gum sandarach for thirty-six hours, a separation might be obtained with much less pain than that occasioned by instant wedging. Thought that a practitioner should form a mental impression of an operation, from its beginning to its completion before its commencement. He considered Dr. Forbes right in his consideration of the comfort and interest of the patient, and would not himself excavate and fill difficult cases at the same sitting.

Dr. Atkinson said that wedging would, from pressure at the apicial foramen and consequent tension of the pulp nerve, in a measure anæsthetize a tooth.

Dr. Parker called attention to a method of keeping the mouth dry, which was certainly new and original. He employs disks of porcelain, which, being placed with the convex surface next the ducts, arrest the flow of saliva, and frequently adhere strongly to the surface of the mucous membrane. He has a number of these plates, as, after being once used, they require heating before suitable for a second application. Between teeth he places a small piece of spunk saturated with perchloride of iron, to prevent the exudation of moisture during the consolidation of the gold.

Dr. Taft spoke of the difference in the character of saliva, and said that he would never perform a submarine operation. He succeeds in keeping the mouth dry by means of bibulous paper placed over the ducts, upon the tongue, and a little roll under the wedge when filling between teeth, renewing the latter occasionally, when necessity required. He employs the double-pronged tongue-holder for retaining the paper in position and preventing interference from the tongue. He uses the warm air blow-pipe for drying the cavity, and believed that the difficulty from rolling or tipping of the gold would never occur if carefully worked from the centre toward the ends. He uses adhesive foil No. 4 almost exclusively, and believed it disadvantageous to make frequent changes, which always necessitate an alteration in the method of operating.

Dr. Robinson packs the walls of a cavity with non-adhesive, and fills in the centre with crystal gold foil.

Dr. J. Ward Ellis would not advocate filling under water, but had met with cases in which he was unable to do otherwise, and had been pleased and surprised to find fillings introduced under such unfavorable circumstances presenting a perfect appearance, even at the expiration of six years. He did not think the profession so wonderfully in advance of twenty years ago, and could not yet regard the mallet or any other peculiar method of manipulation as indispensable to the accomplishment of the best results, for he was frequently seeing *perfect* fillings which had been introduced some twenty-five years ago by hand pressure alone. In filling between molars, he files a V-shaped opening, cuts through the crown of the tooth, and fills perpendicularly downward.

Dr. Perkins fills at the sides and bottom of a cavity with non-adhesive foil, and in the centre with adhesive gold, condensing with the mallet. He cared not what material was employed or how it was manipulated, so that the great desideratum—*the preservation of the tooth*—was accomplished; and referred to filling in his own mouth of twenty years' standing.

Dr. Robinson regarded tin foil as a valuable agent for the preservation of teeth, and instanced a filling of this material which has now stood the test of sixteen years.

Dr. Buckingham regarded tin foil as a very good substitute for gold. He advocated social clinical gatherings of the profession for mutual benefit.

Dr. Burgess believed amalgam applicable in some cases, and instanced several perfect fillings of that material, which he had recently seen, of twenty years' standing.

Dr. Gerry said that if he possessed any hobby, it was surely his uncompromising opposition to amalgam; and when asked the ground of his objection, replied that it was from the fact that mercury is such a powerful oxidizing agent.

The following gentlemen were announced as constituting the Executive Committee for the ensuing year: Drs. Taft, Peirce, Forbes, Robinson, and Atkinson.

Adjourned.

THIRD DAY—*Morning Session.*

Called to order at 8 A. M. Minutes read and approved.

Dr. Taft had used a great many kinds of foil, and manipulated in as many different ways, but now confined himself entirely to adhesive foil, employing the mallet for its consolidation, and believed if compelled to abandon the use of this instrument, it would almost necessitate his ceasing the practice of dentistry. He remarked that care and skill were necessary to obtain a good adaptation to the walls of a cavity with any gold, and thought that each one should operate in the manner by which he can accomplish the best results. He regarded solidity and adaptation as the requisites for a good filling, and believed these points met most thoroughly by the use of adhesive foil, which he considered not only makes the best filling, but enables us to successfully fill cavities where the non-adhesive gold would not be retained. He always pits the bottom of a cavity for anchorage, and in approximal cavities applies his force as near as possible in a line with the axis of the tooth.

Dr. Perkins had seen and could produce many hand fillings which would defy the efforts to force a plugger through their substance. In some cases he regarded the mallet as very useful, while in others he believed it entirely inadmissible, consequently he disliked to hear such sweeping assertions, either in favor of or against any instrument or method of practice, since they give false impressions and are likely to mislead.

Dr. Taft said there were of course exceptional cases, in which the use of the mallet would be inconvenient and unadvisable, as upon the posterior face of a wisdom tooth.

Dr. Atkinson uses the mallet *always*, and referred to the advantage gained in the distribution of force and the ease with which delicate and tender teeth may be filled. He prefers the mallet instruments to have an angle of from twenty-two and a half to forty-five degrees. If the enamel was exceedingly thin, he had filled over paper in order to retain, as near as possible, the natural color of the tooth.

Upon motion, business was suspended for the presentation of some bills, which were ordered to be paid.

A letter from Dr. A. Hill was read by Dr. Allport, in which he regretted his inability to meet with his beloved brethren; and stated that a severe attack of neuralgia had disqualified him for both business and travel. He sent his best wishes, desired to be remembered to all old friends, and wished to impress that necessity and not indifference was the cause of his absence.

The following communication from Dr. S. J. Cobb was read by Dr. Abell:—

Having attended the American Dental Association that met at Niagara Falls, on the 26th ultimo, not as a delegate, but as an interested spectator belonging to the profession, I must say, with all candor and honesty, that I do feel that the three days there spent in that Association was of more interest and value to me than any three days that I have ever spent since I first engaged in the dental profession. I say this, from the fact that it has never been my good fortune before to attend a meeting either of the American Dental Association or the United States Dental Convention. And now, gentlemen, with your consent, I will make one or two suggestions in addition to those I have heard both here (United States Dental Convention, at Detroit) and at the Association at the Falls, upon dental education. And just here, before making any suggestions, I will say that I do most heartily indorse all that I have heard both here and at the Falls, tending to the education not only of dentists, but the people. I am an advocate of education, notwithstanding my own is limited. Possibly that is the reason why I see, feel, and know the importance of it. We want early education. We want it as early as it is possible to obtain it. Viewing the subject in this light, I come to my first suggestion, and that is, we, as teachers, for we are, and if we are not, we should be, notwithstanding I must confess my inability to teach as I ought, or would like, should go where all teachers first go, and that is, to the school-house. There is where the education of man is commenced; and as we wish to reach the masses or the entire people with our profession, we propose going to the commencing place, and there begin with other teachers, and not only begin, but go through with them,—keep pace with them from the simplest to the most scientific and complicated book that is used in school or college. I do not mean to say in this paper what method of instruction we will introduce in school books, but merely mean to advocate the idea, so that we may take it into consideration; call into counsel our entire profession; not only that, but also call in the ablest authors and publishers of the different school books, and we will soon find a way and plan for what we have. I feel assured, gentlemen, that we can, if we will, furnish to the different authors and publishers of school books some of the most important and valuable matter that has ever found place in their works. I feel furthermore assured that there are none save ourselves, that will hail with more delight and pleasure the forthcoming of such valuable instructions, than the authors and publishers of the above-mentioned books. So I would say to one and all, let us go to work and see what we can do. Before closing, I will suggest an idea, in regard to the dental lessons proposed for school books. We may put it in as questions, asked and answered, as in geography, and other primary studies. For instance, the little fellow stands before you. Question. How many teeth have you, or how many constitute the deciduous set? The answer is given. At what age do you get these teeth? Answer. How long does nature design these teeth to serve you? Answered. Is it important that these teeth should be preserved in a healthy condition during this period? Yes. Why? Answered. Can you preserve them in this condition? Answered. How? Answered. And so on, with the second or adult set, and with many lessons of great importance.

We want to give our instructions in a plain, simple, matter-of-fact sort of way. Want to make our dental lessons easily learned, and long remembered. And now, gentlemen, in conclusion upon this subject, I will say, when we have introduced into the different school books that amount of dental intelligence that we ought to have in them, we can promise ourselves, in a fourth or half century, at furthest, that the people, as a mass, will be fully able and competent to appreciate our services as a dental profession. We will then be able to take the elevated and high position that we have long worked for. We may promise ourselves much, in less time than twenty-five years, from this course or method of instruction; but we must not expect too much in a short time; if we do not reap all of the benefits, somebody else will. I am sure we are receiving the benefits of the hard labor of those who have preceded us, and as such we should certainly feel under obligation to do as much for those that follow us.

This being the first time that I have ever had the honor and pleasure of participating in a meeting of this kind, and having had very little or no experience in preparing an article of the kind, I hope to be excused for the shortcomings that may be found therein.

Respectfully,

S. J. COBB.

Dr. Robinson moved that a committee of three be appointed to prepare a dental catechism for introduction into the common school books.

Dr. Abell delivered an earnest and eloquent speech upon the subject of dental education, in the course of which he remarked that, although he would advocate an effort in the direction proposed, he anticipated difficulty in introducing such a book into the schools. He spoke favorably of the course taken by the *People's Dental Journal*, and would suggest, in connection with this plan, the establishment of a high and remunerative scale of prices, as one of the first means for educating the public to an appreciation of their teeth, and elevating the dignity of the profession.

Dr. J. Ward Ellis advocated the principle embodied in the *People's Dental Journal*, but while the cost of this publication, though small, would preclude its introduction among the poorest classes, the influence of a catechism, such as proposed, would make itself felt in the lowliest hovel in the land.

Dr. Robinson's motion was amended, so that the committee should consist of five members, and so adopted.

The following gentlemen were appointed as members of that committee: Drs. Taft, Abell, Spalding, Buckingham, and Robinson.

Dr. Haskell remarked that, by an increase of circulation, the *People's Dental Journal* could be furnished at much lower prices, and stated that the first number of the first volume could be obtained for distribution at the rate of five dollars per hundred.

Dr. B. Wood read a paper upon "The Advancement of the Dental Profession," in which he set forth claims for the recognition of inventors who contribute much toward the progress, value, and elevation of the dental specialty.

The hour of ten having arrived, religious exercises were opened with prayer by Dr. Atkinson, followed by the singing of a hymn, and the reading of the Scriptures by Dr. Taft.

Short, spirited, and *patriotic* addresses were then delivered by Drs. Atkinson and Robinson.

After a prayer by Dr. Kulp, the loyal and earnest strain of remarks was continued by Drs. Taft, Perkins, and Abell. A prayer from Dr. Taft was followed by the Doxology.

The religious exercises having terminated, the regular order of business was resumed, and the next subject taken up for discussion, viz., "The best method of obtaining accurate impressions and models of the mouth."

Dr. W. H. Allen said that when the lower teeth incline toward each other, he takes an impression of the inner surface in wax, allowing it to extend half way over the grinding surfaces; this he removes, varnishes, reintroduces, and completes the outer section in plaster. In ordinary cases he obtains a wax impression first, afterward employing it as a cup for the retention of the plaster.

Dr. Spalding said that for anything like a full denture, he first takes an impression in wax, makes the models, and swages a metallic plate, which, being coated with plaster, he employs as a cup for obtaining the final impression; in lower cases, sometimes covering it merely with a thin coating of wax. Difficulty had been sometimes experienced from the plaster becoming detached from the impression-cup; this, he said, might be obviated by varnishing the metallic surface, and placing cotton upon it before dry; the plaster, when setting in the meshes of the cotton, would be held firmly in place.

Dr. Field employs the same method as Dr. Spalding, with the exception that he punches holes in the plate for the retention of the plaster.

Dr. Kulp has entire and partial impression-cups, and in full cases uses plaster exclusively, while in partial cases he employs wax and paraffine, a material which he regards as particularly applicable where the teeth converge in the manner referred to. As a base for artificial teeth he almost invariably uses rubber.

Dr. Whitney first takes an impression in wax, makes models, and swages a trial-plate; placing wax upon this, he obtains an impression from the plaster model, trims off any superfluous material, ruffles the surface, covers it with a very thin coating of plaster, and procures an accurate and final impression.

Dr. Kulp puts salt into the plaster to hasten setting, pours the water upon it, mixes, and waits until it commences to stiffen before he introduces it into the mouth.

Dr. W. H. Allen thought it proper to sift the plaster into the water while mixing.

Dr. Haskell thought that nothing was gained by going beyond a simple plaster impression. In difficult partial lower cases, he draws before the plaster becomes too solid, and if any pieces become detached, places them in their proper position upon the cup.

Dr. Buckingham spoke of Dr. Wildman's plan of coloring plaster, to render the lines of separation between the impression and model more easily discernible. By experiment, he was forced to believe that the expansion of plaster was not appreciable.

Dr. Haskell also thought that the expansion of plaster was too trifling to merit consideration.

Dr. Perkins could always succeed with the pure, tough, yellow wax; in softening it, he is careful to avoid overheating, which injures its texture, destroying the toughness.

Upon motion, passed to the consideration of "The relative value of different materials as a base for artificial teeth."

Dr. Kulp had used rubber in 2000 cases, his patients preferring it to any other material. He regarded it as a blessing, and thought its opponents were generally those who had no experience with it.

Dr. Perkins thought a vote of censure deserved for such wholesale destruction of the natural teeth, as must have been made for the introduction of so much artificial work. He regarded continuous gum as the acme of mechanical dentistry, gold and silver following in succession. He considered the combination of rubber and gold-wire gauze was the only promising way of using the former material which he had yet seen.

Dr. Kulp explained, that in his section of country the teeth had been ruined by charlatans, and he was now engaged in repairing the damage which they had inflicted.

Dr. Dunn has for the last eight years given porcelain the preference over any other material for entire sets, and would call upon Dr. Hayes to testify with regard to a case which he had inserted in that gentleman's office, and which he had seen sometime after its introduction. For partial and temporary plates he makes use of rubber.

Dr. Hayes testified to the entirely satisfactory nature of the case introduced by Dr. Dunn. For obtaining impressions, he uses a metallic cup, with a perpendicular edge, which he trims out to prevent cutting the membranous reflections and prominent points; this is filled with plaster, allowing it to lap over the edges of the cup, and also clamped at the heel of the plate, in order to prevent shrinking of the impression when it is afterward gently dryed over the flame of a spirit-lamp. After this treatment, it is invested with sand, and a fusible metal, of one-quarter antimony to three-quarters tin, poured upon its surface, giving the male die, from which the female is obtained, by running upon it a mixture of tin and britannia. He could get a better adaptation with gold, and preferred it to any other base, yet thought that entire porcelain and continuous gum were probably the most artistic styles of work, although the additional bulk necessary to insure strength in either case would prove somewhat objectionable.

Dr. Spalding procures dies in the same way as Dr. Hayes, but uses a mixture of tin, antimony, and bismuth, employing the same metal for both sections of the model.

Dr. Hayes said that he added antimony enough to render it, when poured upon a cold surface, capable of being bent a little without fracture.

Dr. Field, in introducing clasps, has them connected to the plate by an intervening standard of metal, thus preventing injury to the teeth by the collection and retention of food.

Dr. Buckingham thought that a perfect fit could be obtained with rubber, and in finishing such cases never interferes at all with the upper surface. He had found rubber very useful for filling under the teeth in gold cases, and also for repairing work mounted upon that metal.

Dr. Perkins thought that the springing of rubber plates was due to their removal from the flasks before cool. He builds well-defined air-chambers upon the plaster model, and has them, with sharply-marked edges, upon the metallic plate. He would prefer never to use clasps, but was sometimes forced to do so against his will.

Dr. Whitney does not use an embracing clasp, but simply solders to the plate two small pieces of gold, which shall extend to and bear upon the tooth at opposing points.

Dr. Knowlton thought that the springing of rubber plates was owing either to undue pressure in bringing the flask together, or the removal of the work before sufficiently cooled. He thought that a great excess of rubber was apt to ruin work, in the effort to force together the sections of the flask.

Adjourned.

THIRD DAY.—*Afternoon Session.*

Dr. Taft recommends to a patient that which he conceives best adapted to the case. He had been in the habit of lining the lingual surface of rubber plates with gold; but intended to try the gold wire or gauze net framework.

The greater part of the afternoon was consumed with a clinical demonstration by Dr. Atkinson.

The following resolution was offered by Dr. Magill:

*Whereas*, In the opinion of this Convention, business will be more promptly accomplished by the preparation of papers upon regular subjects of discussion, rather than by extemporaneous effort;

*Resolved*, That we request of those who intend to meet with us in the next annual convention, to examine carefully the subjects offered, and to the best of their ability prepare well-digested articles, confining themselves carefully to the subjects under discussion, and to forward such papers to be read in Convention, in case they can not attend in person. Carried.

Dr. Allport offered the following:—

*Whereas*, In the opinion of the American Dental Convention, no less than two years pupilage in the office of a competent dentist, and attendance upon two full courses of lectures in a dental college, will qualify an individual to practice dentistry properly; therefore

*Resolved*, That practicing dentists be requested not to receive students into their offices for a less time than two years; and under no circumstances, unless they will agree to attend lectures in and be graduated from a dental college, before entering upon the practice of the profession.

*Resolved*, That the people should require of all those who hereafter enter upon the practice of the profession, that they shall have received a diploma from a dental college as the first requisite for public confidence and patronage. Carried.

Dr. Benedict was glad to have had the Convention honor the City of Detroit with a visit, and hoped to see the State of Michigan well represented at Chicago, in the month of July, 1865.

Dr. Buckingham moved that the Convention extend a vote of thanks to those of the profession of Detroit who have taken an interest in our proceedings. Carried.

Dr. Spalding moved a vote of thanks to the Judge of the Supreme Court, for the privilege of meeting in the room used for the sittings of that honorable body. Carried.

Upon motion, adjourned to meet at White Sulphur Springs, Ohio, on the first Tuesday of August, 1865, at ten A. M.—*Dental Cosmos.*

